# Successful Hybrid Endovascular and Open Approach for Exclusion of a Left Subclavian Artery Aneurysm

**DOI:** 10.3400/avd.cr.24-00139

**Published:** 2025-05-17

**Authors:** Kotaro Mukasa, Yasunori Yakita, Ryosuke Marushima, Shinichiro Abe, Soichi Asano

**Affiliations:** Department of Cardiovascular Surgery, Chiba Cardiovascular Center, Ichihara, Chiba, Japan

**Keywords:** subclavian artery, thoracic endovascular aortic repair, aneurysm

## Abstract

Subclavian artery aneurysms are rare and challenging to treat owing to their anatomical location and proximity to critical branches, including the vertebral artery. We report the case of a 78-year-old man with a left subclavian artery aneurysm. The aneurysm was located in the mediastinum and measured 31 mm in diameter. The proximal side of the aneurysm was sealed with a stent graft, while the distal side was accessed through a supraclavicular incision and directly ligated. Postoperative imaging confirmed complete exclusion. This hybrid approach avoided invasive open surgery and provided a favorable outcome.

## Introduction

Subclavian artery aneurysms are rare but can be challenging to approach owing to their anatomical location. The presence of multiple critical branches, such as the vertebral artery, can complicate endovascular repair. We report the case of a left subclavian artery aneurysm located within the mediastinum that was successfully excluded by terminating blood flow from both the proximal and distal ends through thoracic endovascular aortic repair and simple ligation. The patient provided written consent for publishing the case, including images.

## Case Report

**Figure 1:**
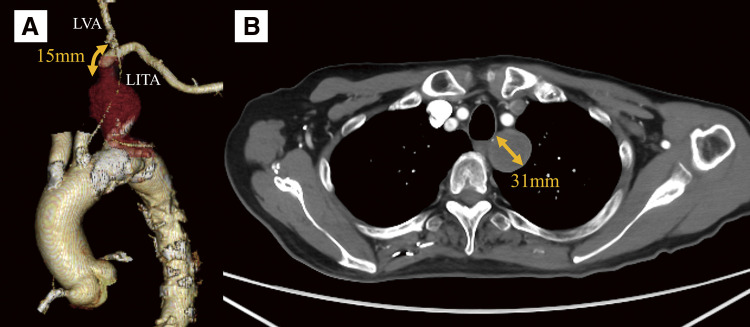
Fig. 1 Preoperative contrast-enhanced computed tomography images. (**A**) Three-dimensional reconstruction image showing the positional relationship between the left subclavian artery aneurysm, the left vertebral artery, and the left internal thoracic artery. To enhance the visualization of the left subclavian artery, the brachiocephalic artery and the left common carotid artery are rendered transparent. (**B**) Axial image. The left subclavian artery aneurysm is occluded by thrombus. LVA: left vertebral artery; LITA: left internal thoracic artery

A 78-year-old man with a history of multiple percutaneous coronary interventions for angina pectoris was incidentally found to have a left subclavian artery aneurysm during computed tomography (CT). Contrast-enhanced CT revealed an aneurysm with a maximum short diameter of 31 mm (**Fig. 1**). The aneurysm extended from the root of the subclavian artery to 15 mm proximal to the left vertebral artery. It was significantly thrombosed, and the left upper limb was perfused via the vertebral artery. Contrast-enhanced CT and magnetic resonance angiography confirmed patency of the vertebral arteries bilaterally, with favorable collateral connection between them. Blood pressure in the left forearm was 40 mmHg lower in systolic pressure and 30 mmHg lower in mean pressure compared with the right forearm. The aneurysm had enlarged by 10 mm over the past 2 years, and owing to the potential risk of rupture, surgery was scheduled. Additionally, the left internal thoracic artery originated more distally than the left vertebral artery.

The surgical plan aimed to exclude the aneurysm by blocking proximal and distal blood flow. The thoracic endovascular aortic repair occluded the proximal side, while the distal side was accessed through a supraclavicular incision and ligated directly. Although subclavian steal syndrome remained a potential risk, preoperative findings indicated that blood flow to the left upper limb was already dependent on the vertebral artery, and the patient exhibited no symptoms during exertion. Therefore, no bypass between the left subclavian artery and other vessels, such as the left common carotid artery, was performed, considering the risks of complications such as stroke associated with carotid manipulation. The distance between the left common carotid artery and the left subclavian artery was 22 mm, which provided an adequate landing zone.

**Figure 2:**
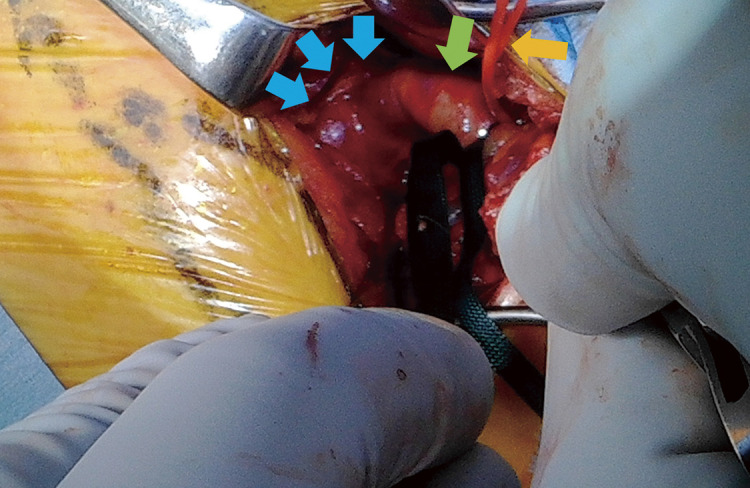
Fig. 2 Intraoperative photography showing the left subclavian artery (green arrow), the proximal aneurysm (blue arrows), and the tape applied around the left vertebral artery (yellow arrow). The upper right corresponds to the cranial side, and the lower left corresponds to the caudal side.

Under general anesthesia, a horizontal 5 cm incision was made in the left supraclavicular fossa following preoperative ultrasound marking of the subclavian artery. The left internal jugular vein and common carotid artery were identified promptly, and the left subclavian artery was exposed. The distal side of the aneurysm was accessed, and the left vertebral artery was identified (**Fig. 2**). The area between the distal end of the aneurysm and the vertebral artery was cleared, and no branches were observed. The artery was clamped between the vertebral artery and the distal end of the aneurysm. After confirming that there was no drop in left upper limb blood pressure and that the vertebral artery was accurately identified, the subclavian artery was ligated at this site. Preoperative contrast-enhanced CT confirmed the absence of thrombus at the ligation site. Subsequently, the thoracic endovascular aortic repair was performed through the right femoral artery, and a Relay Pro 34 × 34 × 145 mm stent graft (Terumo Aortic, Sunrise, FL, USA) was deployed in Zone 2, covering the entry of the left subclavian artery.

**Figure 3:**
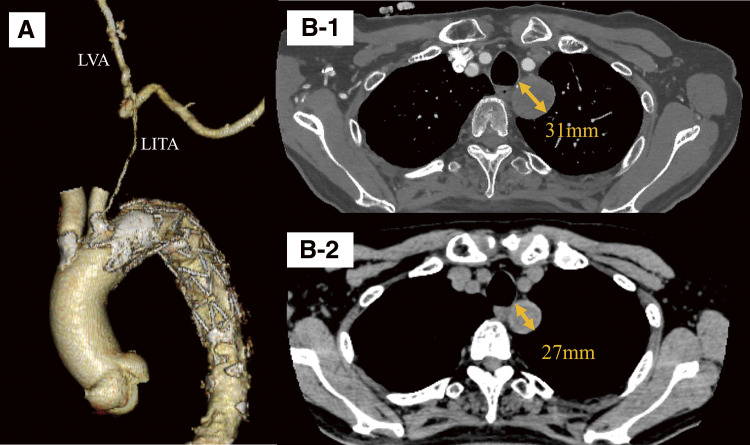
Fig. 3 (**A**) Three-dimensional reconstruction image of the immediate postoperative computed tomography. The left vertebral artery and left internal thoracic artery are preserved. (**B-1**) Axial image from the immediate postoperative period. No obvious endoleak is observed. (**B-2**) Axial image at 3 months postoperatively. The aneurysm has shrunk to 27 mm. LVA: left vertebral artery; LITA: left internal thoracic artery

Postoperative contrast-enhanced CT confirmed successful exclusion of the aneurysm without any blood flow into the sac (**Figs. 3A** and **3B-1**). The patient did not exhibit symptoms suggestive of subclavian steal syndrome during left upper limb exertion postoperatively. While the patient initially experienced dysphonia and mild aspiration during swallowing, these symptoms improved over time. It was suspected that a minor injury to the left vagus nerve occurred during cervical manipulation or the inflammatory response spread locally. At 3 months postoperatively, CT confirmed that the aneurysm had shrunk to 27 mm (**Fig. 3B-2**).

## Discussion

Subclavian artery aneurysms, especially those caused by atherosclerosis, are extremely rare. According to Dent et al., only two cases (0.13%) of subclavian aneurysms were identified of 1488 cases of atherosclerotic true aortic aneurysms.^1)^ By contrast, pseudoaneurysms of the subclavian artery may result from traumatic injuries or inadvertent arterial punctures during central venous catheterization.^2,3)^ The subclavian artery is anatomically divided into proximal, middle, and distal portions based on its relationship with the anterior scalene muscle, with the proximal portion being the most common site for aneurysm formation, accounting for 39% of cases.^4)^ Another classification method differentiates between aneurysms inside and outside the thoracic cavity. The most frequent causes of extrathoracic subclavian aneurysms are trauma and thoracic outlet syndrome, while intrathoracic aneurysms are often owing to atherosclerosis.^5)^

Although ligation of the left subclavian artery may be performed without significant adverse events in many cases, certain risks remain. Weigang et al. reported that covering the origin of the left subclavian artery with a stent graft led to decreased left arm blood pressure in 50% of patients and neurological complications such as brainstem infarction or transient ischemic attacks in 10%.^6)^ A carotid-subclavian bypass can be performed to maintain sufficient blood flow to the left subclavian and vertebral artery territories. However, this procedure is associated with a 2.1% risk of stroke owing to manipulation of the cervical vessels.^7)^ In this case, we chose to avoid bypass surgery, as the patient’s right vertebral artery was not hypoplastic, and there were no clinical signs of subclavian steal syndrome or transient ischemic attacks.

Owing to their anatomical inaccessibility, the intrathoracic location of subclavian artery aneurysms presents a challenge for open surgical repair. Median sternotomy provides limited access to only the root of the left subclavian artery, making exposure to the vertebral artery bifurcation challenging. By contrast, while a supraclavicular incision allows access to the vertebral artery bifurcation, it makes exposure to the subclavian artery root more difficult. Left thoracotomy with the removal of the first rib can provide better exposure,^8)^ but thoracotomy is considered excessively invasive for cases where graft replacement is not required. Therefore, we selected a hybrid approach in which the proximal portion was occluded endovascularly, and the distal portion was directly ligated. Plug embolization was considered for the distal end of the aneurysm. However, the short distance (15 mm) between the distal end of the aneurysm and the left vertebral artery raised concerns about inadvertent vertebral artery coverage. Therefore, we chose direct ligation of the distal end of the aneurysm.

## Conclusion

We performed exclusion of the left subclavian artery aneurysm using thoracic endovascular aortic repair and direct ligation through a supraclavicular incision. Endovascular repair can be challenging owing to the relationship between the subclavian artery and its branches, such as the vertebral artery. Additionally, anatomical factors often make open repair difficult. In such cases, a hybrid approach can be a suitable option.

## Declarations

### Funding

Not applicable.

### Informed consent

Written informed consent was obtained from the patient for the publication of this case report and the accompanying images.

### Acknowledgments

The authors thank Editage (www.editage.jp) for English language editing.

### Disclosure statement

Not applicable.

### Author contributions

Cared for the patient, obtained patient consent, prepared the computed tomography imaging data, and wrote the report: YY, RM, SAb, and SAs

Critical review and revision: all authors

Final approval of the article: all authors

Accountability for all aspects of the work: all authors.
